# Alpha-lipoic acid upregulates the PPARγ/NRF2/GPX4 signal pathway to inhibit ferroptosis in the pathogenesis of unexplained recurrent pregnancy loss

**DOI:** 10.1515/med-2024-0963

**Published:** 2024-06-05

**Authors:** Yan Zhao, Xiaoxuan Zhao, Xiaoling Feng

**Affiliations:** Department of Gynecology, The First Affiliated Hospital of Heilongjiang University of Chinese Medicine, Harbin, 150040, China; Department of Traditional Chinese Medicine (TCM) Gynecology, Hangzhou Hospital of Traditional Chinese Medicine Affiliated to Zhejiang Chinese Medical University, Hangzhou, 310007, China

**Keywords:** unexplained recurrent pregnancy loss, alpha-lipoic acid, ferroptosis, PPARγ/NRF2/GPX4

## Abstract

**Aim:**

With unknown etiology and limited treatment options, unexplained recurrent pregnancy loss (URPL) remains a thorny problem. Ferroptosis, a newly identified type of cell death, has been shown to be crucial in the development in reproductive disorders. This study aims to explore the specific mechanism of ferroptosis in URPL and to uncover whether alpha-lipoic acid (ALA) can inhibit ferroptosis, and then exert a protective effect in URPL.

**Method:**

The decidua tissues of URPL and control patients who actively terminated pregnancy were collected. The CBA/J × DBA/2 murine models of URPL were established, and were randomly treated with peroxisome proliferator activated receptor γ (PPARγ) agonists (Rosiglitazone) and ALA. The CBA/J × BALB/c murine models of normal pregnancy were intraperitoneally injected with PPARγ inhibitors (T0070907). Here, we used reactive oxygen species (ROS), malondialdehyde (MDA), glutathione (GSH)/GSSG, and FeRhoNox-1 analysis to detect the level of ferroptosis. We used quantitative real-time reverse transcription polymerase chain reaction (qRT-PCR) analysis to evaluate the mRNA level of PPARγ. Besides, western blot and immunofluorescence were utilized to test the expression profile of PPARγ/nuclear factor erythroid 2-related factor 2 (NRF2)/glutathione peroxidase 4 (GPX4).

**Results:**

In this study, we found that iron deposition was increased in the decidual tissue of patients with URPL. Additionally, the changes in cell morphology, the level of ROS, MDA, GSH, and the expression of ferroptosis marker proteins NRF2/GPX4 confirmed activated ferroptosis in URPL. Besides, bioinformatics analysis combined with experiments confirmed that PPARγ was critical in triggering NRF2/GPX4 pathway in URPL. Furthermore, URPL mouse models were established, and the results showed that PPARγ/NRF2/GPX4-mediated ferroptosis was also significantly increased, which could be mitigated by ALA treatment.

**Conclusion:**

Overall, these findings suggest that ferroptosis may play an important role in URPL, and ALA might be a promising therapeutic drug for improving pregnancy outcomes in URPL via targeting the PPARγ/NRF2/GPX4 pathway.

## 1 Introduction

Recurrent pregnancy loss (RPL) is usually defined as two or more miscarriages before 20 weeks of gestation [1], which affects approximately 1–2% of reproductive women trying to conceive [2]. Multiple pathogenic mechanisms complicated in RPL have been verified, including uterine abnormalities, endocrinological disorders, genetic anomalies, thrombophilia, and immunological factors [2]. Unfortunately, the causes of half cases, also known as unexplained RPL (URPL), remain poorly understood [3]. Ferroptosis, a newly emerged form of regulated necrotic cell death, is featured as the iron-dependent activation of lipoxygenase and the consequential cell death induced by excessive lipid peroxidation [4]. It has been confirmed that ferroptosis is triggered in a number of diseases and is closely associated with adverse pregnancy outcomes [5,6]. The latest research based on bioinformatics analysis revealed that multiple ferroptosis-related genes were differentially expressed in the decidual tissue of URPL [7]. However, there are still few studies to confirm the role of ferroptosis in the regulation of the pathological mechanism of URPL.

Previous research has identified that almost all genes associated with ferroptosis are mediated by nuclear factor erythroid 2-related factor 2 (NRF2) transcription, including, but not restricted to, glutathione peroxidase 4 (GPX4) [4,8]. NRF2 is a highly sensitive transcription factor to oxidative stress, which can not only facilitate regulation of cellular redox stability but also protect cells from ferroptosis by targeting gene expression involving glutathione (GSH) synthesis and iron metabolism [9]. It has been confirmed that various proteins and enzymes are expressed under the mediation of Nrf2 to prevent lipid peroxidation and subsequent ferroptosis [10]. Of note, GPX4, one of the main downstream target gene of NRF2, is the main inhibitor of ferroptosis by acting as a repair enzyme to catalyze the reduction of lipids and other peroxides [4,11]. So far, research has elucidated that activating the NRF2–GPX4 axis exerts a significant role in preventing lipid peroxidation and ferroptosis [12]. Some evidence to date suggests that ferroptosis may play an important role in UPRL. For instance, serum malondialdehyde (MDA), the oxidation product of polyunsaturated fatty acids, is significantly increased in URPL [13]. However, the NRF2–GPX4 axis has not been discussed in URPL.

Alpha-lipoic acid (ALA), also named as 1,2-dithiolane-3-pentanoic acid, is a biological thiol widely existing in prokaryotic and eukaryotic cells [14,15]. It has been demonstrated to exert several positive effects on human health, such as acting as a biological antioxidant, metal chelator, and detoxifying agent [16]. Besides, ALA also exhibits remarkable anti-inflammatory activities and is supposed to be a possible treatment for a number of inflammatory conditions that disrupt female reproduction [15,17]. To date, various studies have investigated that ALA exerts an evidence-based protective role in RPL [18]. However, the specific mechanisms of ALA by which it protects from early pregnancy loss remain poorly understood. Recently, various studies have investigated that ALA can attenuate ferroptosis via multiple molecular signaling pathways [19]. A study by Zhao et al. confirmed that ALA could alleviate fluoride-induced hepatocyte injury by preventing ferroptosis through the system Xc-/GPX4 axis, lipid peroxidation axis, and iron metabolism axis [20]. Additionally, ALA has been unraveled to alleviate ferroptosis in the MPP(+)-induced PC12 cells via activating the PI3K/Akt/Nrf2 pathway [21]. Moreover, ferroptosis-related signaling proteins targeted by ALA also play an important role in RPL. But whether ALA’s protection against URPL is related to its intervention in ferroptosis has not been studied.

Hence, the present study aimed to investigate the regulatory mechanisms of NRF2/GPX4-induced ferroptosis in URPL, and to explore whether ALA could effectively alleviate ferroptosis and restore impaired reproductive function in URPL.

## 2 Materials and methods

### 2.1 Reagents and antibodies

ALA (T5625) was purchased from Sigma. T0070907 (HY-13202) and rosiglitazone (HY-17386) was purchased from MedChemExpress. GSH and GSSG assay kit (S0053) and MDA assay kit (S0131S) were purchased from Beyotime Biotechnology. FeRhoNox-1 (Fe^2+^ indicator, MX4558) was purchased from Maokangbio. Tissue reactive oxygen species (ROS) test kit was obtained from BestBio (BB-470537). Western blot experiments were performed using the following antibodies: anti-β-actin (4970, 1:1,000) was purchased from Cell Signaling Technology. Anti-peroxisome proliferator activated receptor γ (PPARγ; sc-7273, 1:1,000), anti-NRF2 (sc-365949, 1:1,000), and anti-GPX4 (sc-166570, 1:1,000) were purchased from Santa Cruz Biotechnology. Goat anti-rabbit IgG (H&L) (926-32211, 1:5,000) and goat anti-mouse IgG (H&L) (926-68070, 1:5,000) were purchased from LI-COR. Immunofluorescence experiments were performed using the following antibodies: anti-PPARγ (sc-7273, 1:200), anti-NRF2 (sc-365949, 1:200), and anti-GPX4 (sc-166570, 1:200). Goat anti-mouse IgG H&L (Alexa Fluor® 488) (ab150113, 1:200) was purchased from Abcam. TRIzol reagent (15596026) was purchased from Invitrogen. HiFiScript cDNA Synthesis Kit (CW2569M) was purchased from CWBIO. SYBR Green Pro Taq HS (AG11701) was purchased from AgBio.

### 2.2 Human samples

The decidual tissues were collected after obtaining written informed consent from 15 patients diagnosed with URPL and 15 normal pregnant women at the first affiliated hospital of Heilongjiang University of Chinese Medicine. This project met with the declaration of Helsinki in clinical research and was granted by the human research ethics committee of Heilongjiang University of Chinese Medicine (HZYLLBA2021014). There was no difference in age, gestational period, and menstrual cycle between the two groups of patients. The baseline data of clinical samples are shown in Table A1.

### 2.3 Animals and grouping

A total of 42 CBA/J female mice (6–8 weeks), four 9 BALB/c mice and 12 male DBA/2 mice were obtained from the Beijing HFK Bioscience CO., LTD and housed in a standardized laboratory environment: temperature 22°C, 50–60% humidity, and 12:12 h light–dark cycle with free access to water and food. After 1 week of adaptive feeding, the CBA/J female mice during the proestrus period were randomly divided into two groups, in which the CBA/J × BALB/c combination demonstrated normal pregnancy, whereas the CBA/J × DBA/2 mouse combination exhibited pregnancy loss as an acknowledged model of URPL, which was first proposed by Clark et al. [22]. The mice cohabited at 16:00 each afternoon, and the females were examined at 8:00–8:30 the next day, and finding a vaginal plug under the microscope was regarded as the first day of pregnancy (D1). On D14, mice were anesthetized and sacrificed, and endometrial tissue was collected for subsequent experiments. All of the experimental procedures were supervised by the Institutional Animal Care and Use Committee of Heilongjiang University of Chinese Medicine (Ethics number: 2023042804), and the research was performed in accordance with ARRIVE guidelines 2.0.

To address the role of PPARγ in NRF2/GPX4 pathway in URPL, PPARγ inhibitors (T0070907) and agonists (Rosiglitazone) were utilized, and the mice were divided into the following groups: Control, Control + T0070907, URPL, and URPL + Rosiglitazone group. To address the role of ALA in PPARγ/NRF2/GPX4 pathway in URPL, the mice were divided into the following groups: Control, URPL, and URPL + ALA group. T0070907 was administered once daily by intraperitoneal injection at 5 mg/kg, and rosiglitazone was administered once daily by intraperitoneal injection at 0.1 mg/kg. Moreover, mice in the URPL + ALA group received treatment once daily with ALA (50 mg/kg) by oral administration according to the previous studies [23]. Mice in the control and URPL group were received saline.

### 2.4 GSH/GSSG, MDA, and ROS assays

Decidua GSH content was measured using a GSH and GSSG assay kit (Beyotime, S0053) as per the manufacturer’s instructions. The GSH content of the test samples was calculated as: total GSH–GSSG × 2. Decidua MDA content was measured using a lipid peroxidation MDA assay kit (Beyotime, S0131S) following the manufacturer’s instructions. Moreover, ROS levels in the tissues were detected with tissue ROS detection kit obtained from BestBio Company.

### 2.5 Transmission electron microscope (TEM)

Trypsinase-digested decidual tissue was quickly fixed for 24 h in 2.5% glutaraldehyde solution, washed with PBS, and then transferred to 1% osmium and 1.5% K_3_[Fe(CN)_3_] for 1 h. The decidual tissues were soaked in a dioxy solution of 2% acetic acid at 4℃ overnight, and then dehydrated in a graded ethanol solution and immersed in varying proportions of pure acetone and embedding agent. The samples were thinly sectioned after being polymerized at 60℃. Then the sections were stained using uranyl acetate and lead citrate (3%) solutions. The formation of mitochondria was observed under an electron microscope (Hitachi, Japan).

### 2.6 Fe^2+^ content

The decidual tissues were removed and fixed overnight in 4% paraformaldehyde. Dehydrated with 20–30% sucrose the next day and wait for complete dehydration. The completely dehydrated tissues were prepared into 12 μm frozen sections for immunofluorescence staining. FeRhoNox-1 was placed on the frozen sections and incubated for 60 min at 37°C in a dark chamber. Thereafter, the specimen was counterstained with 4′,6-diaminido-2-phenylindole, dihydrochloride (DAPI) and photographed under a fluorescence microscope (OLYPUMS, Japan).

### 2.7 Quantitative real-time polymerase chain reaction (q-PCR)

q-PCR analysis was conducted following previously described protocols. Total RNA from cells was extracted using TRIzol reagent, and reverse transcription was performed using the HiFiScript cDNA Synthesis Kit. The resulting cDNA was subjected to q-PCR using the SYBR Green Pro Taq HS. The relative mRNA expression level was calculated using the comparative 2^−ΔΔCt^ method based on the threshold cycle (Ct). The primer sequences used for real-time PCR are as follows: PPARγ Forward primer (5′–3′): TCTGGCCCACCAACTTTGGG, PPARγ Reverse primer (5′–3′): CTTCACAAGCATGAACTCCA; GAPDH Forward primer (5′–3′): CAGGAGGCATTGCTGATGAT, GAPDH Reverse primer (5′–3′): GAAGGCTGGG GCTCATTT.

### 2.8 Western blot

Total proteins were extracted with RIPA lysis buffer (Beyotime, P0013B) and protease inhibitors, and the protein concentrations were detected with BCA Protein Assay Kit (Beyotime, P0011). SDS-PAGE was used to separate the proteins that were transferred afterward onto nitrocellulose membranes by wet transfer at 280 mA. The membranes were blocked with 5% nonfat milk for 1 h at room temperature and incubated overnight at 4°C with primary antibodies. Then, the membranes were followed by incubations with secondary antibodies. After the membranes were scanned in Odyssey fluorescence imaging system, quantitative analysis was performed by Image J software.

### 2.9 Immunofluorescence

The frozen sections were blocked with BSA (BSA 5 g, 30%Triton X-100 0.5 mL, sodium azide 0.05 g, 1× PBS) at room temperature for 2 h. Afterward, tissue sections were incubated with primary antibodies at 4°C overnight: anti-PPARγ (sc-7273, 1:200), anti-NRF2 (sc-365949, 1:200), and anti-GPX4 (sc-166570, 1:200), followed by incubation with the secondary antibody: goat anti-mouse IgG H&L (Alexa Fluor® 488) (ab150113, 1:200) in the dark for 2 h. Besides, cell nuclei were stained with DAPI. Finally, images of tissue sections were photographed under a fluorescence microscope (OLYPUMS, Japan). The mean immunofluorescence intensity of PPARγ, NRF2, and GPX4 were performed by Image J software (National Institutes of Health, Bethesda, MD, USA). Three to five different high magnification (×200) fields of the tissue were randomly selected from each section for the statistics of the average optical density value, and the average value was taken as the average optical density value of the section.

### 2.10 Searching and screening microarray data

The microarray datasets were systematically extracted from the gene expression omnibus (GEO) database (https://www.ncbi.nlm.nih.gov/geo/) [24] with the keywords of “recurrent pregnancy loss,” “recurrent miscarriage,” “unexplained recurrent spontaneous abortion,” and “unexplained recurrent spontaneous abortion.” The screening conditions were as follows: (1) *Homo sapiens*, (2) expression profiling by array, and (3) all samples were taken from the endometrial tissue during the mid-secretory phase of menstruation. Finally, two datasets were included (GSE26787 and GSE165004).

### 2.11 Data preprocessing, normalizing, and screening of differentially expressed genes (DEGs)

The background calibration, data normalization, and log2 transformation were conducted on the included data sets using affy in R software (version 4.1.2) [25]. When multiple probes corresponded to one specific gene, their average expression level was considered [26]. Moreover, we used the surrogate variable analysis in Bioconductor to reduce batch effects and other variables [27]. The principal component analysis before and after the batch correction was conducted. At last, the “limma” packages in R software was used to identify the DEGs of RPL with the criteria of adjusted *P*-value <0.05 and |log2 fold change (FC)| > 0.05. DEGs screened from the integrated dataset were shown by heat map and volcano plots.

### 2.12 Acquisition of genes associated with ferroptosis

We obtained the ferroptosis-related genes from the FerrDB database (http://www.zhounan. org/ferrdb)[28], GSEA database (https://www.gsea-msigdb.org/gsea/) [29], and Genecards (https://www.genecards.org/) [30] (screening genes with relevance score ≥1.5), the acquired genes will be combined, and ferroptosis-related genes will be found after removing duplicate genes.

### 2.13 Acquisition and protein–protein interaction (PPI) analysis of URPL-related ferroptosis genes

Intersection genes of URPL DEGs and ferroptosis-related genes were obtained by Venn diagram. Then, we constructed a PPI network of URPL-related ferroptosis genes using the STRING (https://cn.string-db.org/) [31] online website, and the cytoHubba plug-in in Cytoscape software (3.8.1) was used to identify the top ten maximum margin criterion proteins.

### 2.14 Kyoto Encyclopedia of Genes and Genomes (KEGG) functional enrichment analysis of URPL-related ferroptosis genes

In this study, we used the clusterProfiler package for KEGG functional enrichment analysis of URPL-related ferroptosis genes.

### 2.15 Screening the transcription factor of NRF2 in URPL

We performed the PROMO database (https://alggen.lsi.upc.es/cgi-bin/promo_v3/promo/promoinit.cgi? dirDB = TF_8.3) (set the factors predicted within a dissimilarity margin less or equal than 15%) [32] and TRRUST database (https://www.grnpedia.org/trrust/) [33] to find corresponding transcription factor NRF2. The obtained NRF2 transcription factors were intersected with URPL-related ferroptosis genes to identify transcription factors that might regulate NRF2 in URPL.

### 2.16 Statistical analysis

All experiments were repeated at least three times. Data were presented as mean ± standard deviation. All the performed statistical analyses were described in each figure legend. Statistical *P*-values were obtained by application of the appropriate statistical tests using the GraphPad Prism 9. For all tests, *P* < 0.05 was considered significant (*P < 0.05, **P < 0.01).


**Ethical approval:** This study complied with the Declaration of Helsinki in clinical research and was approved by the human research ethics committee of Heilongjiang University of traditional Chinese medicine (HZYLLBA2021014). Written informed consent was obtained from individual or guardian participants. All of the animal experimental procedures were supervised by the Institutional Animal Care and Use Committee of Heilongjiang University of Chinese Medicine (Ethics number: 2023042804), and the research was performed in accordance with ARRIVE guidelines 2.0.

## 3 Results

### 3.1 Increased ferroptosis in decidual tissue in URPL patients

**Figure 1:**
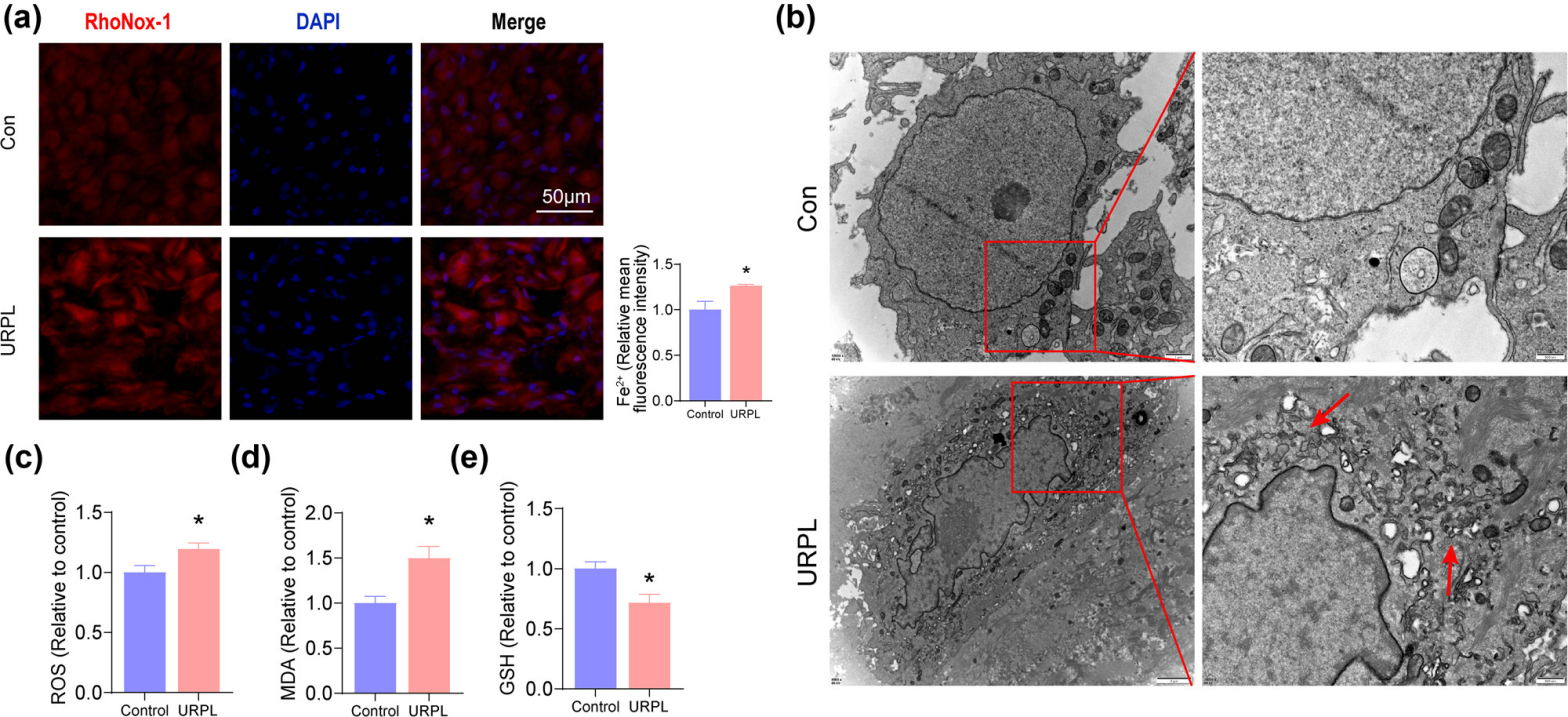
Increased ferroptosis in decidual tissue in URPL patients. (a) Fe^2+^ content was assessed by RhoNox-1 staining. Scale bar: 50 μm. (b) Ferroptosis in decidua was observed by TEM. The red arrow shows a decrease in mitochondrial volume and an increase in cavitation. Scale bar: 2 μm (left), 500 nm (right). (c)–(e). ROS, MDA, and GSH levels were observed by GSH/GSSG, MDA, and ROS assays. *P < 0.05 compared with the control group.

First, we observed Fe^2+^ content in URPL patients and health controls. The RhoNox-1 staining results showed that Fe^2+^ content was increased in the URPL group when compared with the control group (Figure 1a). Moreover, TEM showed that there was a decrease in mitochondrial volume and an increase in cavitation in the URPL group, as compared with the results in the control group (Figure 1b). Next, the relative values of ROS, MDA, and GSH were assessed. Results showed that the ROS and MDA levels were significantly increased, and GSH was significantly decreased in the URPL group (Figure 1c–e).

### 3.2 Expression of NRF2/GPX4 was decreased in URPL

**Figure 2:**
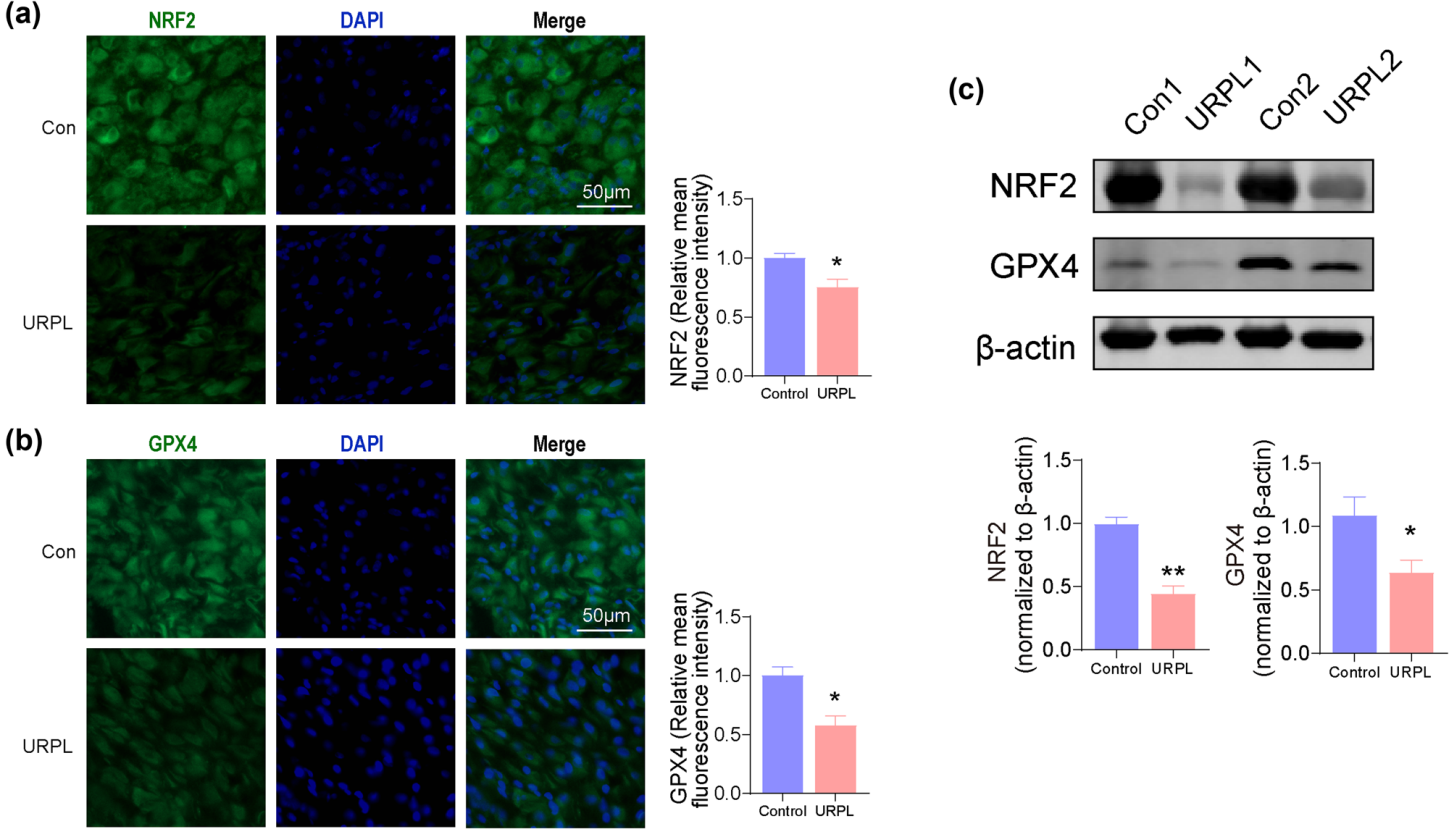
Expression of NRF2/GPX4 in decidual tissue of URPL patients decreased. (a) and (b) NRF2 and GPX4 were assessed by immunofluorescence. Scale bar: 50 μm. (c) NRF2 and GPX4 were assessed by western blot. *P < 0.05, **P < 0.01 compared with the control group.

GPX4 is a phospholipid hydroperoxidase that protects cells against membrane lipid peroxidation. NRF2 can protect cells from ferroptosis by targeting gene expression involving GSH synthesis such as GPX4. The western blot and immunofluorescence showed that the expression levels of the NRF2 and GPX4 significantly decreased in the URPL group, as compared with the results in the control group (Figure 2a–c).

### 3.3 PPARγ-mediated ferroptosis in URPL through NRF2/GPX4

**Figure 3:**
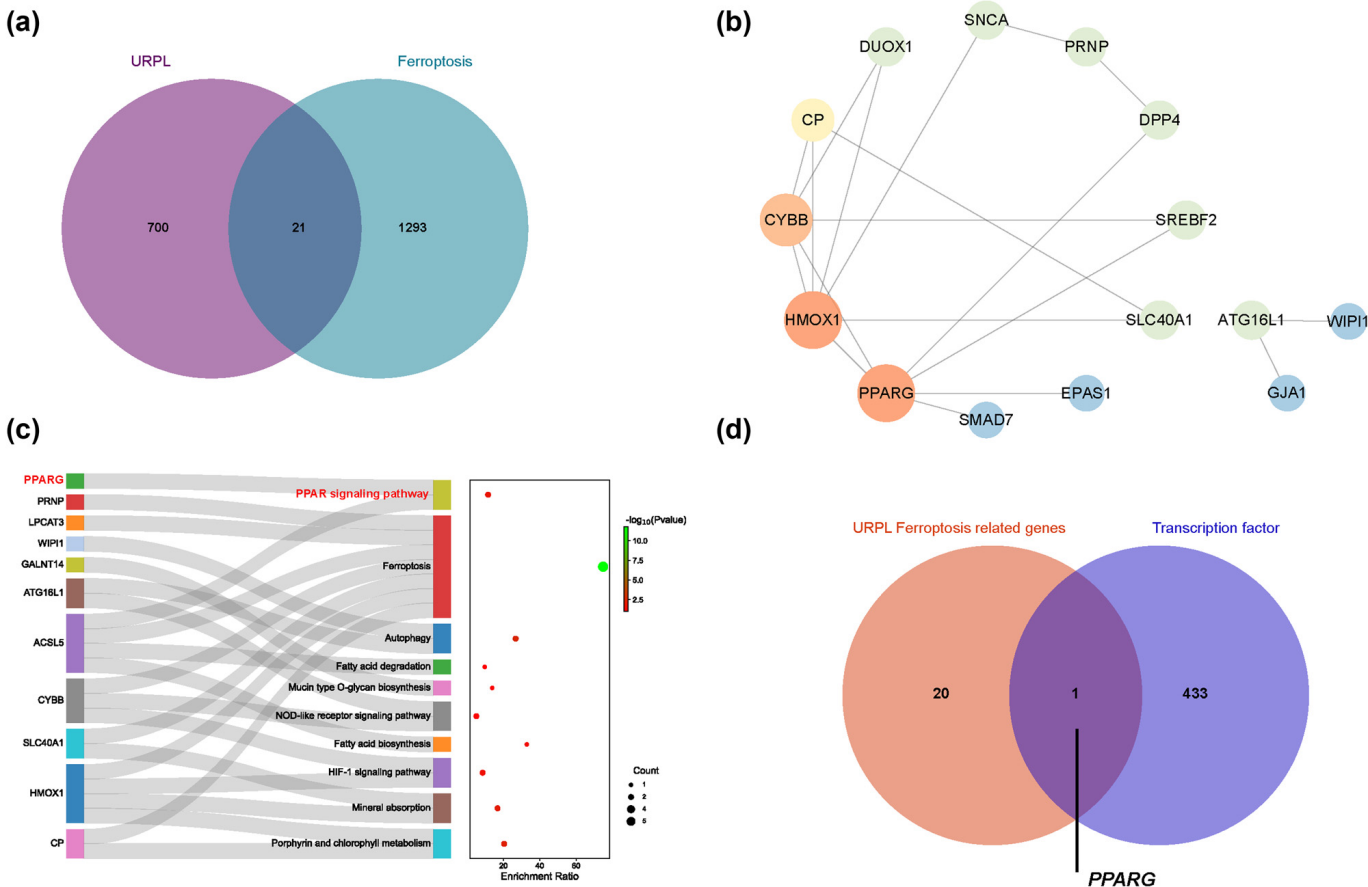
Bioinformatics analysis revealed PPARγ-mediated ferroptosis in URPL through NRF2/GPX4. (a) Venn diagram shows URPL ferroptosis-related genes. (b) PPI analysis of the URPL ferroptosis-related genes. (c) KEGG of URPL ferroptosis-related genes. (d) Venn diagram shows the intersection gene between URPL ferroptosis-related genes and the transcription factor of NRF2.

**Figure 4:**
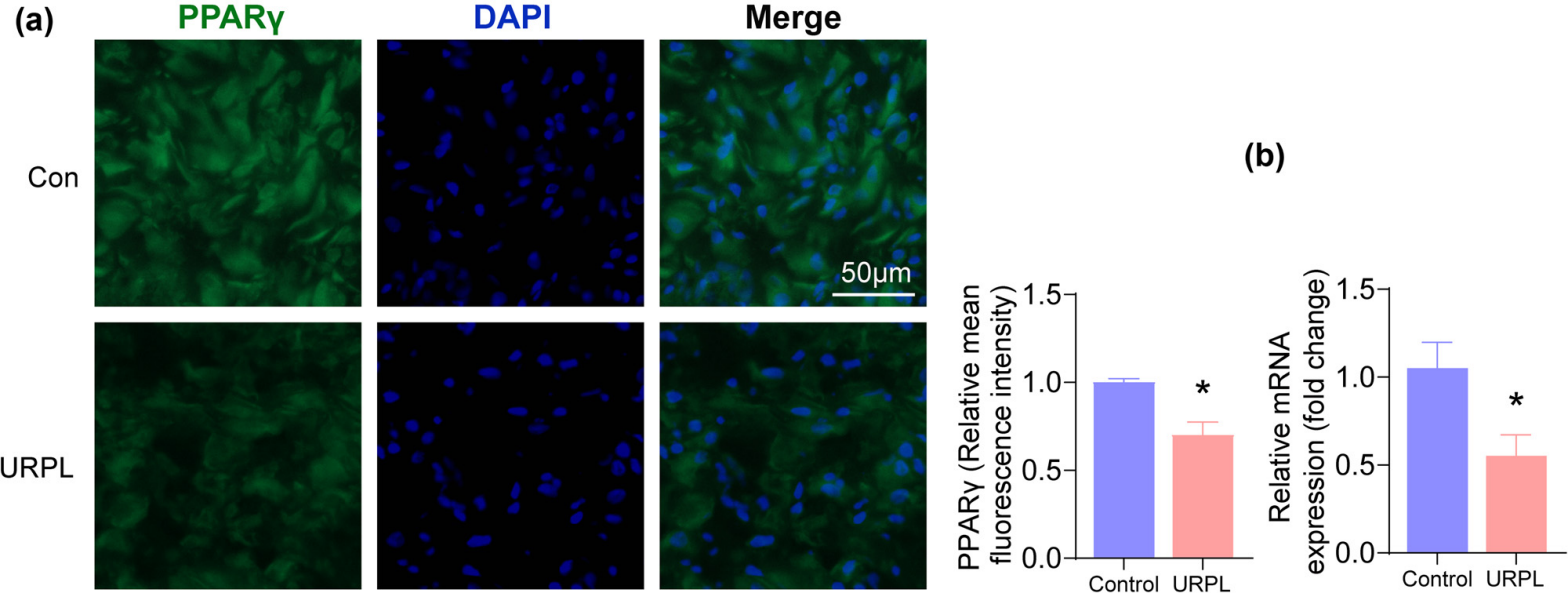
Expression of PPARγ in decidual tissue of URPL patients decreased. (a) PPARγ was assessed by immunofluorescence. Scale bar: 50 μm. (b) mRNA level of PPARγ was assessed by qRT-PCR. *P < 0.05 compared with the control group.

Next, we sought to study which regulated NRF2/GPX4 ferroptosis in URPL via bioinformatics analysis. The results displayed that there were 721 DEGs in URPL. Then 483 ferroptosis-related genes were obtained from FerrDB database, 64 were from the GSEA database and 918 were from Genecards. Subsequently, a total of 1,314 ferroptosis-related genes were obtained by removal of duplicate genes from the three databases. After that, 21 intersection genes of URPL DEGs and ferroptosis-related genes were obtained, namely URPL ferroptosis-related DEGs (Figure 3a). Then, PPI was conducted to reveal inter-molecular interactions during ferroptosis in UPRL. And the results showed that PPARG and HMOX1 were hub genes, and these two proteins were closely correlated with CYBB, DPP4, SREBF2, and SLC40A1 (Figure 3b). Besides, KEGG pathway enrichment analysis displayed that URPL ferroptosis-related DEGs were mainly enriched in ferroptosis, PPAR signaling pathway, fatty acid biosynthesis, and so on. Besides, PPARG was one of the core genes in PPAR signaling pathway (Figure 3c). Furthermore, PPARγ was a key transcription factor of NRF2 associated with ferroptosis in URPL according to the transcription factor analysis based on PROMO and TRRUST databases (Figure 3d). In addition, immunofluorescence revealed that the immunofluorescence intensity of PPARγ was significantly decreased in the decidua of URPL (Figure 4a). Uniformly, the mRNA level of PPARγ was also significantly decreased in the decidua of URPL detected by qRT-PCR (Figure 4b). Thus, PPARG may play an important role in ferroptosis in URPL.

### 3.4 PPARγ/NRF2/GPX4-mediated ferroptosis in URPL

**Figure 5:**
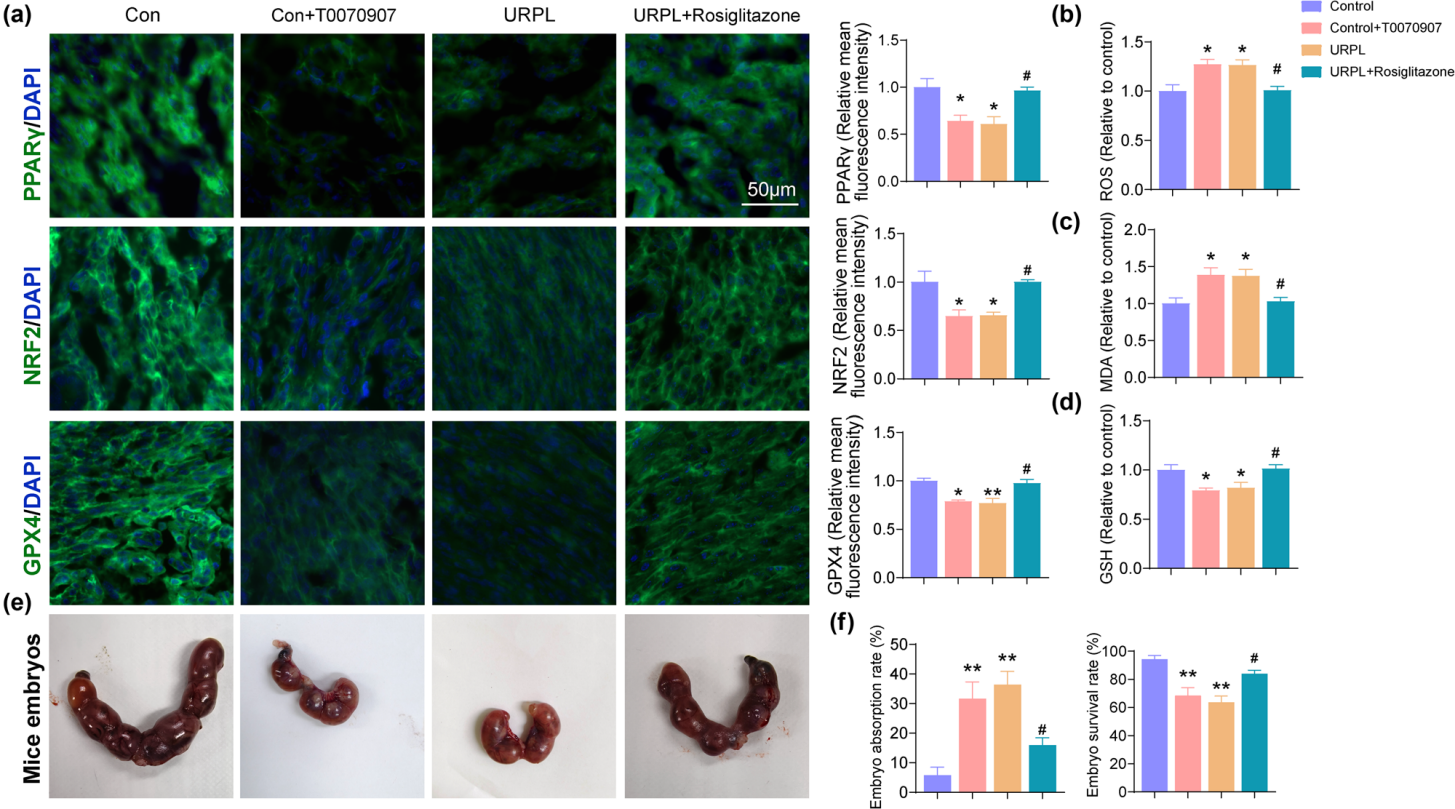
PPARγ/NRF2/GPX4-mediated ferroptosis in URPL. (a) Fe^2+^ content was assessed by RhoNox-1 staining; the PPARγ, NRF2, and GPX4 were assessed by immunofluorescence. Scale bar: 50 μm. (b)–(d) ROS, MDA, and GSH levels were observed by ROS, MDA, and GSH assays. (e) and (f) Absorption of mouse embryos. *P < 0.05, **P < 0.01 compared with the control group, #*P* < 0.05 compared with the URPL group.

To address the role of PPARγ in the NRF2/GPX4 pathway in URPL, we investigated the mechanism of PPARγ/NRF2/GPX4-mediated ferroptosis in URPL by using PPARγ inhibitors (T0070907) and agonists (Rosiglitazone) in mouse models. First, the immunofluorescence intensity of PPARγ/NRF2/GPX4 were significantly decreased in the control + T0070907 and the URPL group, as compared with the control group. Then the immunofluorescence intensity of PPARγ/NRF2/GPX4 were significantly increased in URPL + Rosiglitazone group when compared with the URPL group (Figure 5a). Next, the relative values of ROS, MDA, and GSH were assessed. Results showed that the level of ROS and MDA was significantly increased, and GSH was significantly decreased in the control + T0070907 and the URPL group when compared with the control group. Besides, the level of ROS and MDA was significantly decreased, and GSH was significantly increased in the URPL + Rosiglitazone group when compared with URPL group (Figure 5b–d). Next, we found that inhibition of PPARγ increased embryonic absorption in mice, while activation of PPARγ decreased it (Figure 5e–f).

### 3.5 ALA improved ferroptosis in URPL by activating the PPARγ/NRF2/GPX4 pathway

**Figure 6:**
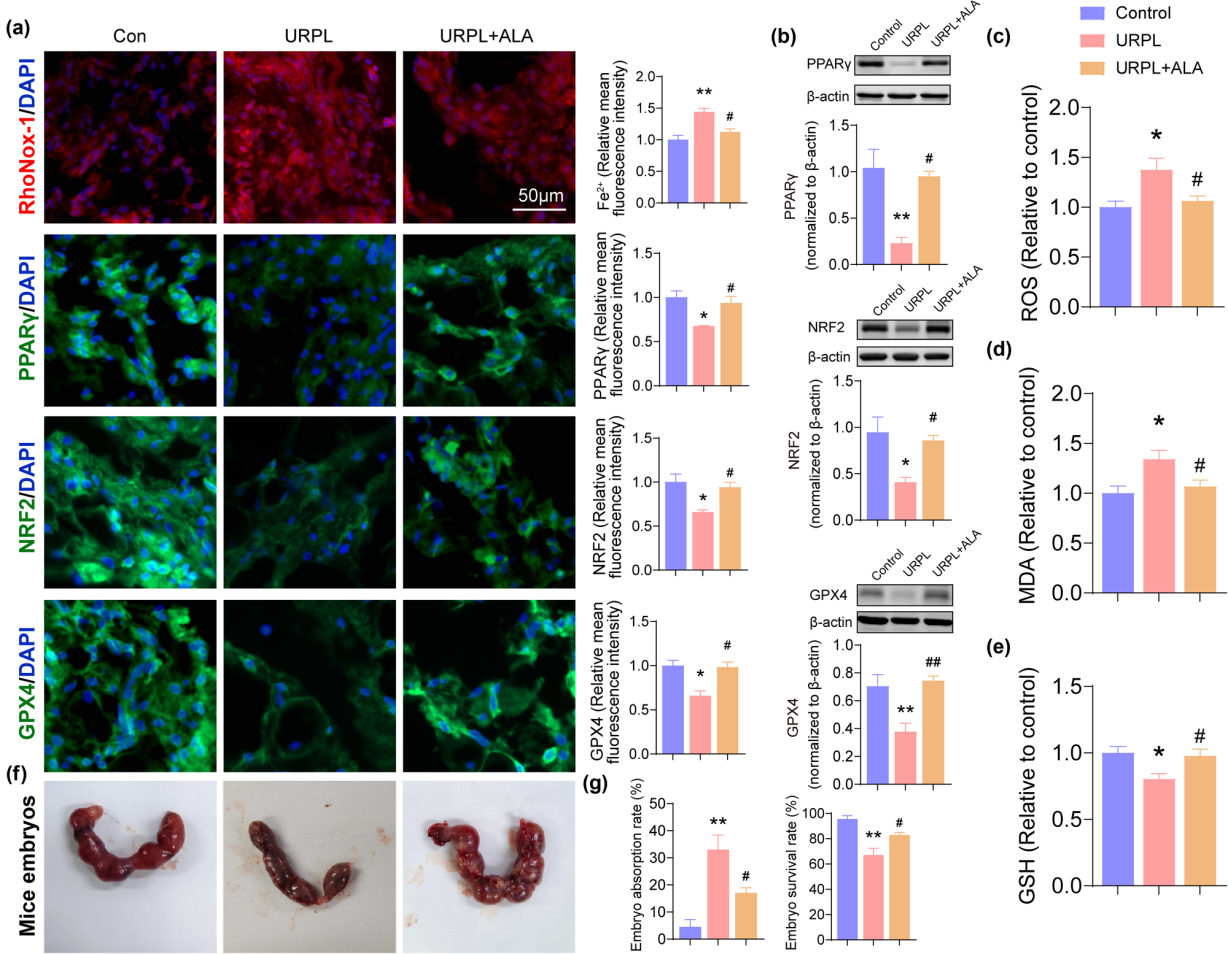
ALA improves ferroptosis in URPL by activating the PPARγ/NRF2/GPX4 pathway. (a) Fe^2+^ content was assessed by RhoNox-1 staining; the PPARγ, NRF2, and GPX4 were assessed by immunofluorescence. Scale bar: 50 μm. (b) PPARγ, NRF2, and GPX4 were assessed by western blot. (c)–(e) ROS, MDA, and GSH levels were observed by ROS, MDA, and GSH assays. (f) and (g) Absorption of mouse embryos. *P < 0.05, **P < 0.01 compared with the control group, #*P* < 0.05 compared with the URPL group, ##*P* < 0.01 compared with the URPL group.

ALA is a widespread organosulfur component with multiple properties. Among them, the strong antioxidant potential makes it a potential drug for patients with threatened miscarriage [18]. First, the Fe^2+^ content was assessed, and the RhoNox-1 staining results showed that the Fe^2+^ content of the URPL group was increased when compared with the control group, and the Fe^2+^ content of the URPL + ALA group was decreased when compared with the URPL group (Figure 6a). Next, the immunofluorescence and western blot showed that the expression levels of PPARγ/NRF2/GPX4 significantly decreased in URPL group, as compared with the results in control group, and the expression levels of PPARγ/NRF2/GPX4 significantly increased in the URPL + ALA group when compared with URPL group (Figure 6a and b). Next, the relative values of ROS, MDA, and GSH were assessed. Results showed that the level of ROS and MDA was significantly increased, and GSH was significantly decreased in URPL group when compared with the control group. The levels of ROS and MDA were significantly decreased, and GSH was significantly increased in the URPL + ALA group when compared with the URPL group (Figure 6c–e). Next, we found that ALA decreased the embryonic absorption in mice (Figure 6f and g).

## 4 Discussion

During pregnancy, the endometrium undergoes significant modifications and evolves into decidua, a newly formed tissue that plays an important part in successful embryo implantation and fetal growth regulation. During this transfer, the endometrium is particularly vulnerable to oxidative stress attack for its high oxygen consumption, active redox-based metabolism, and rich changes in cell fate. Interestingly, oxidative stress has been suggested as a potential mechanism for the occurrence and development of URPL [34]. In this study, we also identified that ROS and MDA levels were significantly increased, and GSH was significantly decreased in the decidual tissue of the URPL group. MDA is one of the products formed by the reaction of lipids with oxygen free radicals, and their content represents the degree of lipid peroxidation. Furthermore, lipid peroxidation caused by oxidative stress is a marker of ferroptosis, which directly damages the integrity of cell membranes and contributes to ferroptotic cell death [35]. Recent studies identify that ferroptosis is closely associated with endometrial-related reproductive disorders. According to Ni et al., iron-rich follicular fluid enhanced the likelihood of endometriosis-related infertility [36]. Besides, Hu et al. discovered a link between uterine and placental ferroptosis and fetal loss caused by oxidative stress [37]. Furthermore, iron deposition and ferroptosis in the decidua have been verified to induce pregnancy loss. And the ferroptosis inhibitor could effectively reverse theembryo loss in the abortion model [38]. In this study, we found that iron deposition was increased in URPL. Data have confirmed that iron overload can facilitate ferroptosis by creating ROS via the Fenton chemistry [39]. Accordingly, we found that ferroptosis was significantly increased in decidual tissues of patients with URPL, characterized by classic ferroptosis changes in cell morphology and cell compositions. Physiologically, decidual tissue is not only to provide a physical anchorage but also to provide a conducive biochemical microenvironment for embryo implantation and development at the early stage, such as immune regulation, vascular recasting embryo implantation, and placentation, thus orchestrating the homeostatic balance between the mother and fetus [40,41]. While, excessive ferroptosis not only causes functional decline or loss but also emphasizes inflammatory signals and promotes embryo rejection [9]. Compelling evidence indicates that ferroptosis holds great potential for initiating inflammation or at least has proinflammatory effects [4]. Inhibiting ferroptosis can prevent the ensuing immune cell invasion and inflammatory reaction, thus contributing to the improvement of various inflammatory diseases [42,43]. Previous advances in immunologic studies have confirmed the overactive inflammation in decidual tissue of URPL, accompanied by high level of proinflammatory cytokines and inflammasome [44]. Besides, there is some preliminary evidence showing that ferroptosis could be important in inducing cell death by immune cells [45]. Similarly, a bioinformatic study has shown that ferroptosis is also associated with immune landscape disorders in the decidual tissue of the URPL [7]. Collectively, ferroptosis and the biological processes secondary to ferroptosis are closely related to the recognition and tolerance of the decidual tissue to the embryo in URPL. However, the specific molecular network responsible for ferroptosis regulation remains to be explored, which may provide potential targets for future treatment.

Previous research has shown that NRF2/GPX4 controls nearly all ferroptosis-related genes. NRF2 exerts significant roles in establishing a cellular antioxidative defense system via regulating iron metabolism, GSH regulation, NADPH regeneration, which is pivotal for GPX4 activity [46,47]. GPX4 is one of numerous members of the GPX family, and it is crucial to ferroptosis development. If GPX4 activity declines, lipid peroxides cannot be broken down by the GPX4-catalyzed reduction process. Additionally, Fe^2+^ oxidizes lipids in a Fenton-like way, producing numerous ROS in the process that encourages ferroptosis. Yang et al. confirmed that cells with downregulated GPX4 expression were more sensitive to ferroptosis, while cells with upregulated GPX4 expression could avoid ferroptosis [48]. So far, studies have shown that the inhibition of NRF2/GPX4 that fails to suppress ferroptosis may be a critical pathomechanism for triggering pregnancy complications such as preeclampsia [49]. However, it is still unclear how NRF2/GPX4 contributes to URPL. In this study, we found that the NRF2/GPX4 signal axis was significantly suppressed in UPRL. Of note, this is the first time that NRF2/GPX4-mediated ferroptosis has been discovered in decidua samples of UPRL. Activating this signal axis may provide a possibility for improving decidual tissue function in URPL.

Despite the fact that NRF2/GPX4 is largely important for preventing ferroptosis, little is known about how cells control their anti-ferroptotic potential in both physiological and pathological conditions. In this study, we confirmed that PPARγ was significantly reduced in the decidua of URPL based on bioinformatics analysis and animal experiments, which may be account of the disregulation of NRF2/GPX4-dependent ferroptosis in URPL. PPARγ belongs to the transcription factor family, and has been demonstrated to orchestrate lipid and glucose metabolism pathways, including lipogenesis, steroidogenesis, and glucose transporters [50]. Previous studies have confirmed that PPARγ can regulate sequences of many genes involved in lipid and glucose metabolism and cell differentiation by binding with retinoid X receptor as a heterodimeric partner to specific DNA sequences, termed PPAR response elements [51]. In addition, PPARγ is highly expressed in the maternal–fetal interface during pregnancy and supposed to represent a critical link between energy metabolism and reproduction [52]. Substantial studies have confirmed that PPARγ can affect trophoblast proliferation, differentiation, and migration via regulating cellular metabolism [53,54]. Similarly, a study by Duan et al. confirmed that the lack of PPARγ could lead to embryonic lethality due to implantation defects [55]. Furthermore, due to its crucial role in antioxidant defense and redox balance [56], recent research begins to highlight the tight link between PPARγ and ferroptosis. A study conducted by Chen et al. displayed that PPARγ stimulated the NRF2/ARE axis to neutralize oxidative stress and ferroptosis [57]. Thus, moving from evidence to practice, our study focused on the modulation between PPARγ and NRF2–GPX4 axis. The result uncovered that PPARγ activation significantly induced NRF2/GPX4 signal, and the level of ferroptosis and the embryo absorption rate in URPL mice were significantly reduced. On the contrary, PPARγ inhibition significantly suppressed NRF2/GPX4 axis and increased ferroptosis and embryo absorption in normal mice. These results suggested that PPAR played a crucial role in NRF2/GPX4-mediated ferroptosis in UPRL. Thus, we presume that PPARγ is a potential target in URPL on account of its ability to attenuate oxidative stress and ferroptosis. Of note, exploring the protective role of PPARγ agonists in URPL is a very promising area. Consistent with our ideas, Rosiglitazone, an agonist of PPAR, has been verified to improve the endometrial receptivity via restraining endometrial angiogenesis during decidualization, while decidualization deficiency is a critical pathomechanism in URPL [58,59]. Besides, in our study, we confirmed that Rosiglitazone significantly ameliorated oxidative stress and upregulated the PPARγ/NRF2/GPX4 signaling axis, which in turn amplified ferroptosis and reduced embryo absorption in mice. However, the safety and efficacy of Rosiglitazone in URPL remains to be validated by clinical data.

Now it has been discovered that iron chelators (Deferoxamine) and lipid peroxidation inhibitors (Ferrostatin-1) can prevent ferroptosis [60]. However, their safety for use in pregnant women has not been confirmed. Therefore, there is an urgent need to explore safe and effective drugs to intervene in ferroptosis in URPL. ALA has been described as an ideal or universal antioxidant in various diseases. Recently, ALA has been suggested as a possible therapeutic approach for several oxidative stress disorders that affect pregnancy outcomes [61]. Its supplementation has been recently proposed in some clinical trials to identify the efficacy in RPL. For instance, a clinical trial studying the effects of ALA supplementation for male partners of couples with RPL showed that ALA mitigates sperm DNA damage and lipid peroxidation in the male partner of couples with PRL [62]. In addition, existing studies have shown that ALA not only has no adverse effects on female and male reproductive function, but also protects oocytes and sperm from toxic substances [63]. In particular, ALA has a protective effect on embryos in adverse environments [64]. Thus, in terms of efficacy and safety, ALA exhibits remarkable potential in the treatment of URPL.

At present, the role of ALA in women with URPL mainly focuses on its anti-inflammatory activity, especially its inhibition of NF-κB. It has been confirmed that ALA can target NF-κB by regulating upstream kinases such as MAPK to prevent IkB degradation, or through regenerating vitamin E and thus inhibiting protein kinase C, which can phosphorylate IkB [15]. Besides, Di Nicuolo et al. showed that ALA supplementation for 3 months significantly reduced the endometrial inflammasome NALP-3 expression and the consequent pro-inflammatory cytokines (IL-18 and Il-1β) secretion in women with RPL [18]. In addition to that, recent studies have proved that ALA could inhibit ferroptosis-like cell death [14,65]. The mechanism study identified that ALA could prevent ferroptosis through the system Xc-/GPX4 axis, lipid peroxidation axis, and iron metabolism axis [21]. Nevertheless, few studies have discussed whether ALA attenuates ferroptosis in URPL. In combination with the latest frontiers, we found for the first time in this study that ALA could improve ferroptosis by acting on PPARγ–NRF2–GPX4 in decidual tissue and significantly reduce abortion in URPL mice. In line with our results, recent investigations indicate that ALA can activate PPAR-γ, modulate PPAR-regulated genes, and upregulate the expression of PPARγ mRNA and protein [66]. Taken together, these findings imply that PPARγ activation by ALA plays a crucial protective role in URPL via targeting ferroptosis.

## 5 Conclusion

In summary, to the best of our knowledge, this is the first time we have identified the role of PPARγ/NRF2/GPX4-mediated ferroptosis in the pathology of URPL, and it is also the first time we have demonstrated that ALA plays a protective role in URPL by regulating ferroptosis via targeting PPARγ/NRF2/GPX4. Taken together, our study throw new mechanistic insights into ferroptosis caused by PPARγ/NRF2/GPX4 in URPL, as well as the experimental and theoretical basis for the treatment of URPL by ALA treatment.

## Abbreviations

ALA: alpha-lipoic acid
DEGs: differentially expressed genes
GSH: glutathione
GPX4: glutathione peroxidase 4
KEGG: Kyoto Encyclopedia of Genes and Genomes
MDA: malondialdehyde
NRF2: nuclear factor erythroid 2-related factor 2
PPARγ: peroxisome proliferator activated receptor γ
PPI: protein–protein interaction
RPL: recurrent pregnancy loss
ROS: reactive oxygen species
TEM: transmission electron microscope
URPL: unexplained recurrent pregnancy loss

## Appendix

**Table A1:** The baseline data of clinical samples

	Control group (*n* = 15）	URPL group (*n* = 15）	*P*
Age (years)	31.60 ± 3.11	31.00 ± 2.95	0.592
The gestation period (days)	58.67 ± 2.69	58.53 ± 3.14	0.901
The menstrual cycle (days)	29.13 ± 2.23	29.00 ± 2.24	0.871
